# Longitudinal Associations of the Cystic Fibrosis Airway Microbiome and Volatile Metabolites: A Case Study

**DOI:** 10.3389/fcimb.2020.00174

**Published:** 2020-04-28

**Authors:** Andrea Hahn, Katrine Whiteson, Trenton J. Davis, Joann Phan, Iman Sami, Anastassios C. Koumbourlis, Robert J. Freishtat, Keith A. Crandall, Heather D. Bean

**Affiliations:** ^1^Division of Infectious Diseases, Children's National Health System, Washington, DC, United States; ^2^Department of Pediatrics, George Washington University School of Medicine and Health Sciences, Washington, DC, United States; ^3^Center for Genetic Medicine Research, The Children's Research Institute, Washington, DC, United States; ^4^Department of Molecular Biology and Biochemistry, University of California at Irvine, Irvine, CA, United States; ^5^School of Life Sciences, Arizona State University, Tempe, AZ, United States; ^6^Center for Fundamental and Applied Microbiomics, The Biodesign Institute, Arizona State University, Tempe, AZ, United States; ^7^Division of Pulmonary and Sleep Medicine, Children's National Health System, Washington, DC, United States; ^8^Division of Emergency Medicine, Children's National Health System, Washington, DC, United States; ^9^Computational Biology Institute and Department of Biostatistics & Bioinformatics, Milken Institute School of Public Health, George Washington University, Washington, DC, United States

**Keywords:** cystic fibrosis, microbiome, metabolome, pulmonary medicine, pediatrics

## Abstract

The identification of 16S rDNA biomarkers from respiratory samples to describe the continuum of clinical disease states within persons having cystic fibrosis (CF) has remained elusive. We sought to combine 16S, metagenomics, and metabolomics data to describe multiple transitions between clinical disease states in 14 samples collected over a 12-month period in a single person with CF. We hypothesized that each clinical disease state would have a unique combination of bacterial genera and volatile metabolites as a potential signature that could be utilized as a biomarker of clinical disease state. Taxonomy identified by 16S sequencing corroborated clinical culture results, with the majority of the 109 PCR amplicons belonging to the bacteria grown in clinical cultures (*Escherichia coli* and *Staphylococcus aureus*). While alpha diversity measures fluctuated across disease states, no significant trends were present. Principle coordinates analysis showed that treatment samples trended toward a different community composition than baseline and exacerbation samples. This was driven by the phylum Bacteroidetes (less abundant in treatment, log_2_ fold difference −3.29, *p* = 0.015) and the genus *Stenotrophomonas* (more abundant in treatment, log_2_ fold difference 6.26, *p* = 0.003). Across all sputum samples, 466 distinct volatile metabolites were identified with total intensity varying across clinical disease state. Baseline and exacerbation samples were rather uniform in chemical composition and similar to one another, while treatment samples were highly variable and differed from the other two disease states. When utilizing a combination of the microbiome and metabolome data, we observed associations between samples dominated *Staphylococcus* and *Escherichia* and higher relative abundances of alcohols, while samples dominated by *Achromobacter* correlated with a metabolomics shift toward more oxidized volatiles. However, the microbiome and metabolome data were not tightly correlated; examining both the metagenomics and metabolomics allows for more context to examine changes across clinical disease states. In our study, combining the sputum microbiome and metabolome data revealed stability in the sputum composition through the first exacerbation and treatment episode, and into the second exacerbation. However, the second treatment ushered in a prolonged period of instability, which after three additional exacerbations and treatments culminated in a new lung microbiome and metabolome.

## Introduction

Cystic fibrosis (CF) is a genetic disease affecting more than 30,000 people in the United States, associated with both intermittent and chronic suppurative lung infections (Wagener et al., 2013; MacKenzie et al., 2014). For the past 15 years, 16S rDNA amplicon sequencing has been a commonly used research methodology to describe the microbiota within the CF airway (Rogers et al., 2004; Harris et al., 2007; Guss et al., 2011). While most studies are cross-sectional, a small number of longitudinal studies have been performed to determine if unique microbial signatures correspond with clinical state before and after the onset of a pulmonary exacerbation, a clinical disease state associated with increased respiratory symptoms, increased airway inflammation, and decreased pulmonary function (Zhao et al., 2012; Carmody et al., 2013; Zemanick et al., 2013). These longitudinal studies have revealed that each CF subject harbors a distinct microbial community, making broad interpretations across persons with CF that could be used in clinical practice elusive (Caverly and LiPuma, 2018). More recent studies have employed unbiased whole genome shotgun sequencing techniques, which can provide more granular information, including species, and strain specificity (Moran Losada et al., 2014; Bacci et al., 2017; Feigelman et al., 2017; Hahn et al., 2018a).

Current clinical practice relies on culturing pathogens from sputum samples, however, culture-independent approaches such as the 16S rDNA amplicon sequencing described above have revealed that CF airways are dominated by bacteria from two main populations: slow-growing opportunistic pathogens (e.g., *Pseudomonas aeruginosa, Stenotrophomonas maltophilia, Burkholderia cepacia* and others), and anaerobes common to the oral cavity that migrate to the airways in the context of poor mucociliary clearance (e.g., *Streptococcus* spp., *Rothia mucilaginosa* and others). Metabolomics offers another culture independent approach to profile clinical samples. A few studies investigating different classes of metabolites from CF breath and sputum have emerged, although it is difficult to determine whether the molecules have human or microbial origin. One study using parallel breath metabolomics and shotgun sequencing found that a microbial fermentation product, 2,3-butanedione, was associated with *Streptococcus* spp. and *Rothia mucilaginosa*, and became less abundant after treatment for pulmonary exacerbation (Whiteson et al., 2014). These data, in combination with studies that are identifying volatile metabolite signatures associated with common CF pathogens in culture (Bean et al., 2016; Bos et al., 2016; Phan et al., 2017; Nasir et al., 2018) and the host's immune response to infection (Bean et al., 2015), suggest that there may be changes in the volatile metabolome of an exacerbation that correspond to microbiome changes. The volatile metabolome may be able to complement genomics as an additional culture-independent method for identifying exacerbation onset and recovery, and/or understanding the root causes of these events.

These factors influenced our initial motivation to identify associations between bacterial genera and volatile metabolites within CF sputum that tracked with variations in clinical state (baseline, exacerbation, and treatment). The objective of this study was to identify the level of intra-person variability between clinical states by deeply examining a single person with CF as a case study that could inform study design in a larger cohort of study participants. We hypothesized that specific associations between bacterial genera and volatile metabolites would be reflective of changes between clinical states.

## Materials and Methods

### Setting and Study Population

This is a study of a single young child with cystic fibrosis (homozygous F508del) with respiratory samples and clinical data collected across five acute pulmonary exacerbations over a 12 month period. The samples used in this study were from a larger prospective, longitudinal study conducted at Children's National Health System (CNHS) (Hahn et al., 2019). The study was approved 02/20/15 by the Institutional Review Board (Pro5655) at CNHS. Parental consent was obtained for the study participant prior to respiratory sample collection and extraction of data from electronic medical records.

### Subject Encounters

Respiratory samples and clinical data were collected at three research encounter types. The first encounter occurred when the study participant was being seen for a regular clinic visit and had not received intravenous (IV) antibiotics for at least 30 days prior (Baseline, B) (Zhao et al., 2012). The next encounter occurred when the study participant presented for a sick visit with at least 4 of 12 Fuchs criteria present and hospital admission for administration of IV antibiotics was required (Exacerbation, E) (Fuchs et al., 1994). The third encounter type occurred while the study participant was on antibiotic therapy (Treatment, T). A new baseline sample was obtained at the study participant's next follow up visit, at least 30 days after completion of antibiotic therapy. However, if the study participant's next exacerbation occurred prior to the 30 day window, the time point was treated as an exacerbation. During each encounter, a sputum respiratory sample was obtained and corresponding clinical data were collected. Lung function testing was collected clinically following ATS spirometry guidelines and were reported using NHANES III reference values (National Center for Health Statistics, 2001; Miller et al., 2005).

### Respiratory Sample Collection, Storage, and Processing

Sputum samples were obtained from the study participant using sterile collection cups and were stored at 4°C for up to 24 h prior to processing. Sputum samples were homogenized in the following manner: washed 1:1 v/v with sterile normal saline, mixed 1:1 v/v with dithiothreitol (DTT; Sputasol, Fisher Healthcare, Houston TX), vortexed, and heated in a 37°C heated water bath for 10 min. Samples were then pelleted through centrifugation (12,000 g × 10 min). Supernatants were removed and stored at −80°C until they were analyzed for volatile metabolites. Cell pellets were frozen at −80°C until they underwent DNA extraction.

### DNA Extraction

Sample pellets were thawed and a 500 μL combination of lysozyme (20 mg/mL, Sigma-Aldrich, St. Louis MO) and lysostaphin (200 μg/mL, Sigma-Aldrich, St. Louis MO) was applied to chemically lyse the bacterial cell walls. DNA extraction was then performed using the DSP Virus/Pathogen Midi Kit and the Complex800_V6_DSP protocol on the QIAsymphony SP (Qiagen, Valencia CA).

### Next Generation Sequencing and Bioinformatics

Sequencing of all samples was performed by the University of Michigan Host Microbiome Initiative (Ann Arbor MI) according to published protocols (Kozich et al., 2013; Seekatz et al., 2015). Briefly, extracted DNA was amplified for the V4 region of the 16S rRNA gene (16S rDNA) using PCR primers (forward primer GTGCCAGCMGCCGCGGTAA; reverse primer TAATCTWTGGGVHCATCAGG) (Kozich et al., 2013). The PCR cycle was as follows: 95°C × 2 min (1x); 95°C × 20 s, 55°C × 15 s, 72°C × 5 min (30x); 72°C × 10 min (1X); 4°C (until sequencing). Libraries were normalized using SequalPrep Normalization Plate Kit (Life Technologies, Carlsbad CA), and concentration measured using Kapa Biosystems Library Quantification Kit (Kapa Biosystems, Wilmington MA). A MiSeq sequencing platform was used to perform the dual-index sequencing strategy, resulting in paired-end reads of ~250 basepairs (bp). Raw FASTQ files were processed in mothur (version 1.39.5), utilizing default settings outlined on the MiSeq SOP (https://www.mothur.org/wiki/MiSeq_SOP, accessed 8 FEB 2018) to generate operational taxonomic unit (OTU) tables (Schloss et al., 2009; Kozich et al., 2013). Specifically, this included the following steps: (1) combining paired reads (quality scores per base had to be > 25 minimum for paired gap sequence and 6 points better than paired mismatch; otherwise the consensus base was set to an N); (2) removing sequences > 275 bp; (3) combining duplicate sequences; (4) aligning sequences to the SILVA_v123 bacterial reference alignment (obtained from http://www.mothur.org); (5) removing sequences outside the expected alignment region of V4 (position 1968 to 11550); (6) pre-clustering sequences differing by 2 bp; (7) removing chimeras; (8) removing non-bacterial sequences, including those that align to Chloroplasts, Eukaryotes, or Archaea; and lastly (9) clustering into OTUs at the 0.03 threshold (97% sequence similarity).

The 16S rDNA sequence files were also analyzed using DADA2 to establish amplicon sequencing variants (ASVs) (Callahan et al., 2016). Prior to importing our sequences into R, FlexBar 3.4 was used to trim adapters and low quality sequences (Roehr et al., 2017). Imported sequences underwent further quality trimming using functions within DADA2 (fastqPairedFilter). Trimmed amplicon sequences were denoised (function dada) and chimeras were removed (function isBimeraDenovo). Taxonomic classification was performed by aligning denoised sequences to an RDP dataset (https://benjjneb.github.io/dada2/training.html), with the taxonomy assigned based on the least minBoot bootstrap confidence.

For 3 sputum samples, shotgun whole genome sequencing (WGS) was also performed at the New York Genome Center (New York NY) using the X10 (Illumina, San Diego CA). WGS libraries were prepared using the Illumina TruSeq Nano DNA Library Preparation Kit in accordance with manufacturer's instructions. Briefly, 100 ng of DNA was sheared using the Covaris LE220 sonicator (adaptive focused acoustics). DNA fragments underwent end-repair, bead-based size selection, adenylation, and Illumina sequencing adapter ligation. Ligated DNA libraries were enriched with PCR amplification (using 8 cycles). Final libraries were evaluated using fluorescent-based assays including PicoGreen (Life Technologies) or Qubit Fluorometer (invitrogen) and Fragment Analyzer (Advanced Analytics) or BioAnalyzer (Agilent 2100). Libraries were sequenced on an Illumina HiSeq X sequencer (v2.5 chemistry) using 2 × 150 bp cycles. FASTQ files were screened for quality using FastQC (bioinformatics.babraham.ac.uk/projects/fastqc/) and trimmed using FlexBar 3.4 (Roehr et al., 2017). OTU tables were created using Pathoscope 2.0, which includes a step for filtering human DNA contamination (Hong et al., 2014). The reference database was created using sequences identified in the National Center for Biotechnology Information (NCBI) Bacteria and Virus reference and representative genome database. This database contains at least one genome for each species in the Entrez genome collection that has assembly data. In addition, we also added complete genome assemblies for all *Pseudomonas aeruginosa, Burkholderia cepacia*, and *Burkholderia cenocepacia*, allowing for strain-specific identification of these species.

KneadData (http://huttenhower.sph.harvard.edu/kneaddata) was used to remove human DNA contamination prior to performing functional analyses. The presence of antibiotic resistance genes was detected using MEGAres and AMRPlusPlus using a Galaxy pipeline (Lakin et al., 2017).

### Volatile Metabolite Analysis

For volatile metabolomics analysis, the sputum sample supernatants were thawed overnight at 4°C, vortexed for ~2 s to homogenize, and 250 μl of the samples were transferred into 2 mL GC vials with PTFE silicone caps. The vials and caps had been heat treated for 24 h at 100°C to reduce the contribution of exogenous volatile organic compounds to the samples. Technical duplicates were prepared for all samples, and the samples were maintained at 4°C until analysis, which was completed within 48 h of removing the samples from the −80°C freezer.

For metabolomics analysis, the order of analysis for the samples and technical duplicates was randomized. Prior to sample analysis, the mass spectrometer was calibrated using perfluorotributylamine. Sputum samples were incubated at 50°C for 2 min with agitation, then the volatile metabolites were sampled with heating and agitation for 10 min via headspace solid phase microextraction (HS-SPME) using a 1 cm triphase fiber (divinylbenzene/carboxen/polydimethylsiloxane, 50/30 μm; Supelco/Millipore Sigma). The volatile molecules were desorbed for 180 s at 250°C, and injected splitlessly onto a comprehensive two-dimensional gas chromatograph with a time-of-flight mass spectrometer (GC × GC-TOFMS; Pegasus 4D, Leco Corp.), equipped with an autosampler (MPS Robotic, Gerstel, Inc.). The volatile metabolites were separated on a two-dimensional column set of an Rxi-624Sil (60 m × 250 μm × 1.4 μm; length × internal diameter × film thickness; Restek) first column and a Stabilwax (1 m ×25 0 μm × 0.55 μm; Restek) second column, joined by a press-fit connection. The primary oven was initialized at 35°C with a 0.5 min hold, then heated at 5°C/min to a final temperature of 230°C (5 min hold). The secondary oven and modulator temperatures were maintained with +5°C and +20°C offsets, respectively, relative to the primary oven. The quad-jet modulator was operated with a 3 s modulation period (0.75 s hot and cold pulses). The helium carrier gas flow rate was 2 ml/min. The transfer line and ionization source temperatures were 250°C. Compounds were ionized by electron impact at −70 eV and mass spectra were acquired with unit mass resolution at 100 Hz over a range of 29–400 *m/z*.

Retention indices (RI) for the sputum metabolites were calculated using external alkane standards (C_6_-C_15_), which were analyzed by HS-SPME GC×GC-TOFMS. The standards were heated to 50°C for 2 min with agitation, then sampled for 2 min using a 1 cm triphase SPME fiber. The alkanes were desorbed from the fiber for 180 s at 250°C and injected with a 50:1 split. All other chromatography and mass spectrometry parameters were the same as for the sputum samples. RIs for sputum volatile compounds eluting prior to hexane or after pentadecane were extrapolated.

Metabolomics data collection, processing, and alignment were performed using ChromaTOF with Statistical Compare, version 4.71 (Leco Corp.). The baseline was drawn through the middle of the noise and the signal-to-noise (S/N) cutoff for initial peak finding was 50 for a minimum of two apexing masses. Subpeaks were combined when the second dimension retention time decrease was ≤ 100 ms and the mass spectral match score was ≥ 500. Peaks were aligned across chromatograms when the first dimension retention time shift was ≤ 3 s (one modulation period) and the mass spectral match score was ≥ 600. A second round of peak discovery was performed on the aligned chromatograms, adding peaks with S/N ≥ 10 if the peak was present in at least one chromatogram with a S/N ≥ 50.

Suspected contaminants, chromatographic artifacts (e.g., atmospheric gasses, siloxanes, phthalates), and peaks eluting prior to acetone at 358 s, which were poorly modulated, were removed from peak tables prior to statistical post-processing. The arithmetic means of technical duplicates were calculated and used for subsequent analyses.

Compounds were assigned to the following chemical classes based upon their second-dimension retention times and matches to the mass spectrum and retention index data in the NIST 14 library: hydrocarbons, alcohols, ethers, aldehydes, ketones, carboxylic acids/esters, aromatics, or sulfur-containing. If more than one functional group was present on a molecule, then the compound was assigned to the chemical class of the highest oxidation state.

### Statistical Analysis

Continuous variables were compared using *t*-test while categorical variables were compared using Chi-square or Fisher's exact test. Richness and alpha diversity was measured by the number of observed OTUs, the Shannon Index and the inverse Simpson's Index using Explicet v.2.10.5 (Robertson et al., 2013; Wagner et al., 2018). OTU and taxonomy tables were imported into Rstudio for subsequent analyses using the packages *randomForest* v.4.6-14, *DESeq2* v.1.24.0, and *phyloseq* v.1.28.0 to classify samples based on a forest of trees, determine differential abundance, and create principle coordinates analysis (PCoA) plots, respectively (Liaw and Wiener, 2002; McMurdie and Holmes, 2013; Love et al., 2014). Permutational multivariate analysis of variance (PERMANOVA) was also calculated to measure the significance of differences in overall bacterial distribution using the *adonis* function of the Rstudio package *vegan* v.2.5–5 (Oksanen et al., 2017).

The metabolomics data were normalized using probabilistic quotient normalization (Dieterle et al., 2006), and both the metabolomics and genomics data were log_10_ transformed. The reads for OTUs assigned to *Escherichia* and *Staphylococcus* were summed to create a single variable, referred to as “normal pathogens.” Manhattan distances between samples were calculated using standardized variables (OTUs and/or chemical classes) using the Rstudio package *factoextra* v.1.0.5.

## Results

### Clinical Course

A young school-age child experienced five exacerbations over 12 months. The child was considered to have an advanced disease stage (with a forced expiratory volume in 1 s, FEV1, < 40%) and severe disease aggressiveness (with an FEV1 < 80% at age < 10 years) (Konstan et al., 2009). Past history included multiple acute pulmonary exacerbations where respiratory cultures had grown many antibiotic resistant organisms, including the following: methicillin resistant *Staphylococcus aureus* (MRSA), *Pseudomonas aeruginosa, Stenotrophomonas maltophilia, Achromobacter xylosoxidans*, and an extended beta-lactamase (ESBL) producing *Escherichia coli*, among others. At study enrollment, the child was receiving thrice weekly azithromycin, inhaled tobramycin (TOBI) every other month, and was alternating oral linezolid and trimethoprim-sulfamethoxazole (trim-sulfa) at two week intervals with a two week break in between. Sputum was collected from the subject at 14 time points over the 12 month study, encompassing baseline (B), exacerbation (E), and treatment (T) periods. Antibiotics during the study were selected by the clinical team, but were typically geared toward treatment of the ESBL *E. coli* using a carbapenem, specifically meropenem or imipenem (E1–E5) and amikacin (E1, E3–E5), and MRSA with linezolid (E1, E2, and E5). The clinical characteristics surrounding each sample collection during the study period are shown in Table 1.

**Table 1:** Study participant clinical characteristics.

**Sample ID, Day sample obtained**	**Clinical culture results**	**Suppressive antibiotics (inhaled or oral)**	**IV Antibiotics given for pulmonary exacerbation**	**Days of IV antibiotic therapy**	**FEV1 (percent predicted)**
B-1 Day 0	MSSA, *E. coli*	Azithromycin, TOBI ON	NA	NA	31
E-1 Day 28	*E. coli*	Azithromycin, TOBI OFF	Meropenem, amikacin, linezolid	20	26
T-1 Day 37	NA	NA			41
B-2 Day 77	Normal respiratory flora	Azithromycin, linezolid, TOBI OFF	NA	NA	36
E-2 Day 84	MRSA, *E. coli*	Azithromycin, linezolid, TOBI OFF	Piperacillin-tazobactam, imipenem-cilastatin, linezolid	13	36
T-2 Day 93	NA	NA			43
E-3 Day 132	MRSA, *E. coli*	Azithromycin, trim-sulfa, TOBI ON	Meropenem, amikacin	29	Not obtained
T-3 Day 148	NA	NA			35
B-4 Day 189	MRSA, *E. coli*	Azithromycin, trim-sulfa, TOBI OFF	NA	NA	27
E-4 Day 210	Normal respiratory flora	Azithromycin, trim-sulfa, TOBI OFF	Meropenem, amikacin	29	28
T-4 Day 225	NA	NA			30
E-5 Day 273	MSSA, *E. coli, A. xylosoxidans*	Azithromycin, TOBI OFF	Meropenem, amikacin, linezolid	26	Not obtained
T-5 Day 284	NA	NA			47
B-6 Day 343	*A. xylosoxidans*	Azithromycin, trim-sulfa, TOBI OFF	NA	NA	35

*FEV1, forced expiratory volume in one second, MSSA, methicillin-sensitive Staphylococcus aureus; MRSA, methicillin-resistant S. aureus; NA, not available; TOBI ON/OFF, inhaled tobramycin on or off; trim-sulfa, trimethoprim-sulfamethoxazole*.

### Taxonomic Profile and Microbial Diversity

**Figure 1:**
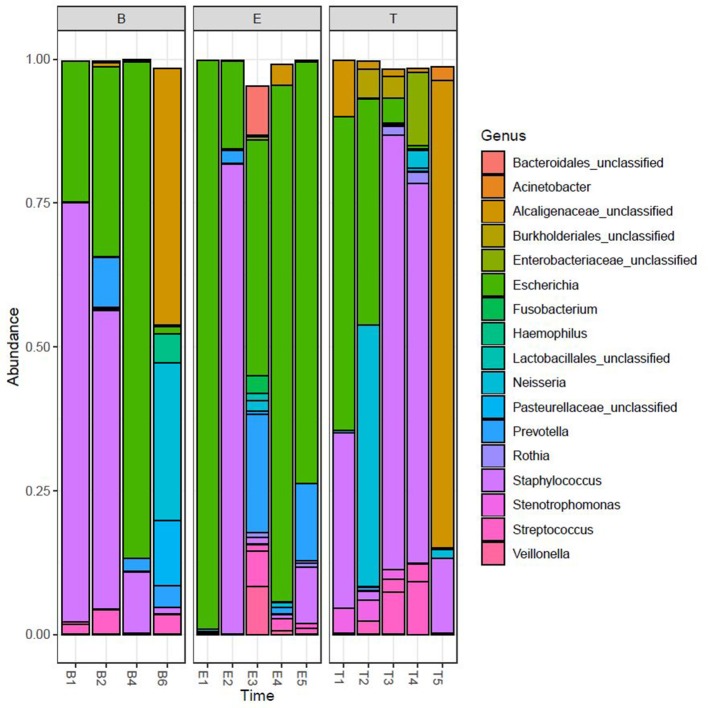
Relative taxonomic abundance. Relative abundance as determined by operational taxonomic units (OTUs). The top 20 OTUs are included. B, Baseline; E, Exacerbation; T, Treatment.

**Figure 2:**
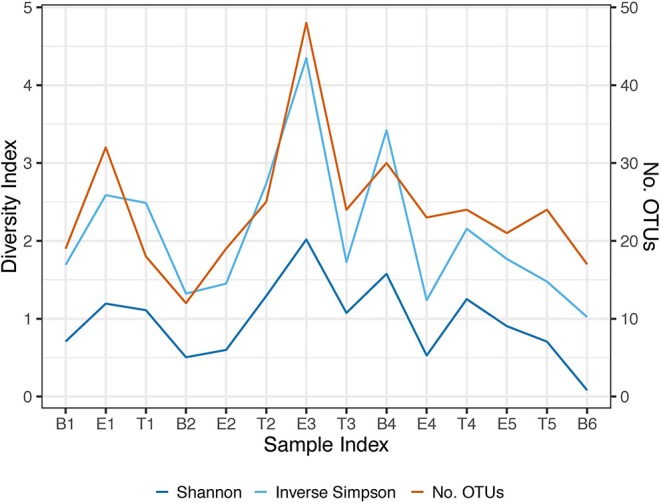
Longitudinal representation of alpha diversity measures. Number of observed OTUs, Shannon Index, and the Inverse Simpson's Index are included. The right axis represents the values of the number of observed OTUs. The left axis represents the values of the Shannon and Inverse Simpson's index. B, Baseline; E, Exacerbation; T, Treatment.

We performed Next Generation 16S rDNA sequencing on the 14 sputum samples. Across all samples there were 109 individual OTUs identified, with individual samples averaging 24 OTUs (standard deviation (SD) 8.3, range 12–48). Three genera dominated the samples, *Escherichia, Staphylococcus*, and *Achromobacter/Alcaligenes* (Figure 1), corresponding to the three most commonly and abundantly cultured pathogens during the study period (Table 1). Alpha diversities were calculated to evaluate the balance of the number of bacteria identified and their relative abundance to each other (Figure 2). The average Shannon diversity index was 0.967 (SD 0.479, range 0.083–2.017) and the average inverse Simpson's index was 2.102 (SD 0.899, range 1.022–4.345). Linear regression was performed to determine if the three dominant genera across samples explained the change in alpha diversity measures over time. No significant differences were found in the number of OTUs, Shannon diversity (*R*^2^ = 0.132, *p* = 0.687), or Inverse Simpson diversity (*R*^2^ = 0.135, *p* = 0.678).

**Figure 3:**
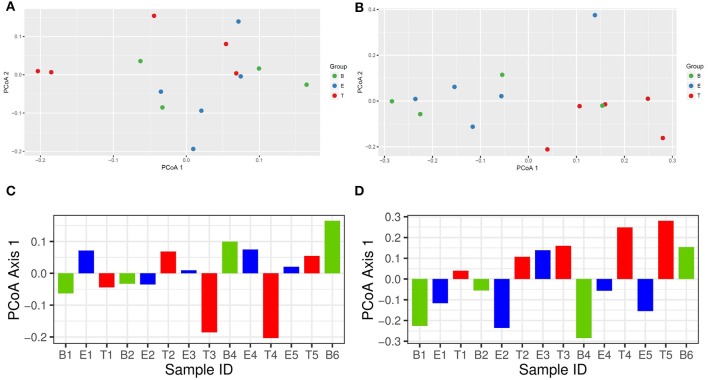
Bray-Curtis distance PCoA with log transformed counts. **(A)** Phylum Axis 1 vs. Axis 2. **(B)** OTU Axis 1 vs. Axis 2. **(C)** Phylum Axis 1 by Sample ID. **(D)** OTU Axis 1 by Sample ID.

To better understand differences in overall community composition between the samples, we evaluated Bray-Curtis distances. The PCoA plots for Axis 1 vs. Axis 2, as well as the variation between samples on Axis 1 alone, are shown in Figure 3. There was no significant difference based on encounter type (baseline, exacerbation, or treatment) when evaluating by phyla or OTU using PERMANOVA (*p* = 0.760 and *p* = 0.433, respectively). However, the treatment samples clustered together when evaluating by OTU (Figure 3B), and warranted further investigation.

**Figure 4:**
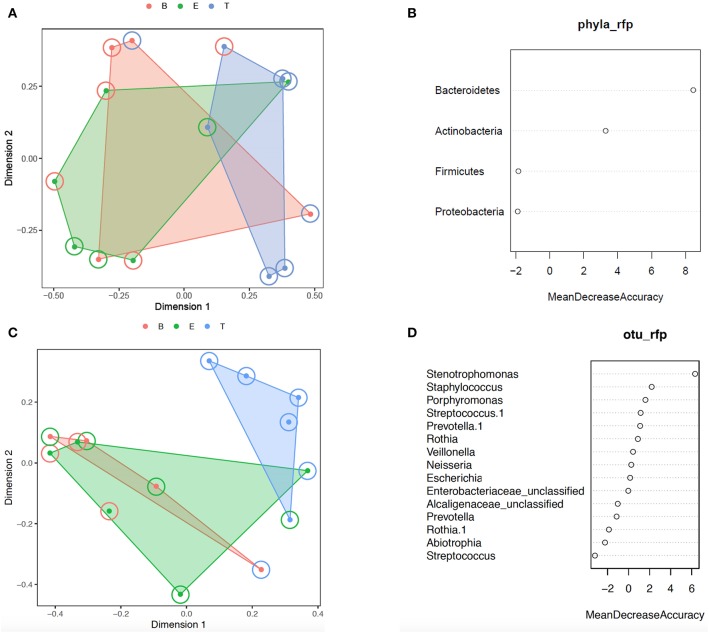
Random forest analysis. **(A)** Phylum proximity plot by encounter type. **(B)** Variable importance plot for Phylum. **(C)** OTU proximity plot by encounter type. **(D)** Variable importance plot for OTU. B, Baseline (red); E, Exacerbation (green); T, Treatment (blue).

Additional analysis of the bacterial phyla and genera present by encounter type was performed using Random Forest. Prior to analysis, the phyla and OTU were assessed to determine prevalence across samples, and those with a prevalence of < 50% were not included in subsequent analysis. This left 4 of 6 phyla and 15 of 109 OTUs as variables. In the analysis of phyla, 500 trees were added with 2 variables tried at each split. A proximity plot from one of the analyses showed significant overlap of encounter types when evaluating by phyla present (Figure 4A), and the out of bag (OOB) estimate of the error rate was 64.3%. The variable importance for phyla present within encounter type was evaluated. Bacteroidetes had a mean decrease accuracy of 8.46 and a mean decrease Gini of 3.34 and Actinobacteria had a mean decrease accuracy of 3.29 and a mean decrease Gini of 1.97, with an OOB estimate of the error rate of 57.1% (Figure 4B). For the analysis by OTU, 500 trees were added with 3 variables tried at each split. The proximity plot for this analysis showed the treatment samples were more distinct (Figure 4C); however, the OOB estimate of error rate remained high at 64.3%. The variable importance was also evaluated for the OTUs present per encounter type. *Stenotrophomonas* was associated with a mean decrease accuracy of 6.33 and a mean decrease Gini of 1.09, with an OOB estimate of the error rate of 64.3% (Figure 4D).

We next evaluated for a differential abundance of certain phyla or OTUs based on encounter type using *DESeq2*. Similar to what we found in the Random Forest analyses, Bacteroidetes were less abundant in treatment samples compared to baseline and exacerbations samples (log_2_-fold difference −3.29, adjusted *p* = 0.015). Also corroborating our Random Forest analyses, *Stenotrophomonas* was more abundant in treatment samples compared to baseline or exacerbation samples (log_2_-fold difference 6.26, adjusted *p* = 0.003).

We also evaluated our 16S rDNA sequencing data by determining amplicon sequencing variants (ASVs). We found a similar relative abundance of the most dominant genera using both methods (Supplementary Figure 1 and Supplementary Table 1). Therefore, we felt confident in using our OTU generated results for subsequent comparisons to our volatile metabolomic data.

Three samples (B1, E3, and E5) also underwent shotgun whole genome sequencing, allowing for additional analyses. These results are included in the Supplementary Data. Briefly, we found a significantly different abundance of the primary bacterial pathogens (*Escherichia coli, Staphylococcus aureus*, and *Achromobacter xylosoxidans*) in the shotgun sequencing data compared to the 16S sequencing data (Supplementary Figure 2 and Supplementary Table 1). We also found 30 different types of bacteriophages within these samples, with the majority of the bacteriophages identified being associated with *Staphylococcus* (*n* = 15, 50%) and Enterobacteria/*Escherichia* (*n* = 12, 40%), which were the predominant bacteria within the microbiome community (Supplementary Figure 3). Lastly, we found a higher percentage of sequencing reads mapped to an antibiotic resistance gene in the exacerbation samples compared to the baseline sample (Supplementary Figure 4). The majority of antibiotic resistance genes identified were due to multi-drug resistance mechanisms (e.g., porins and efflux pumps), while the next most common were aminoglycosides, beta-lactams, and fluoroquinolones. These findings corroborated the antibiotic resistance recognized in the corresponding clinical cultures and suggest shotgun metagenomics provides deeper insights into the microbiome diversity as well as antibiotic resistance.

### Volatile Metabolite Analysis

We analyzed the headspace volatiles of the sputum samples using comprehensive two-dimensional gas chromatography–time-of-flight mass spectrometry (GC×GC-TOFMS). After data alignment and artifact removal, we detected 460 non-redundant volatile metabolites from the fourteen sputum samples, all of which were detected in at least two specimens, with a median frequency-of-observation (FOO) of 12 specimens. While we expect that the dithiothreitol treatment of the sputum altered the metabolome of the samples, e.g., affecting the relative abundances of sulfurous compounds, all sputum samples were similarly processed, and thus we assumed that comparative analyses between baseline, exacerbation, and treatment samples were not impacted. Since our hypothesis for this study was that the volatile metabolomes would change with clinical disease state, we reduced our list of variables to those in the top 50th percentile for variance across samples (calculated as the relative standard deviation, with missing values replaced with zeros), yielding 229 volatile metabolites for subsequent analyses with a median FOO of 8 specimens. We tested for associations of specific volatile metabolites to disease state using pair-wise comparisons of ET vs. B, EB vs. T, and E vs. TB. We did not identify any analytes that were significantly different using Wilcoxon signed rank with Benjamini-Hochberg correction (α = 0.05). We also performed supervised Random Forest using the same categories, with 100 models each initialized with 499 trees. We built models using all observations, as well as using balanced class sizes created by down-sampling. Based on error estimates averaged over all trees, the resulting models were overfit, with out of bag class errors exceeding 60% in all models. The feature set of individual metabolites is sparse (i.e., many missing values), which we posited was contributing to the overfitting. Therefore, for subsequent analyses, we used chemical classes for correlation analyses, reducing the number of features by two orders of magnitude with no missing values.

**Figure 5:**
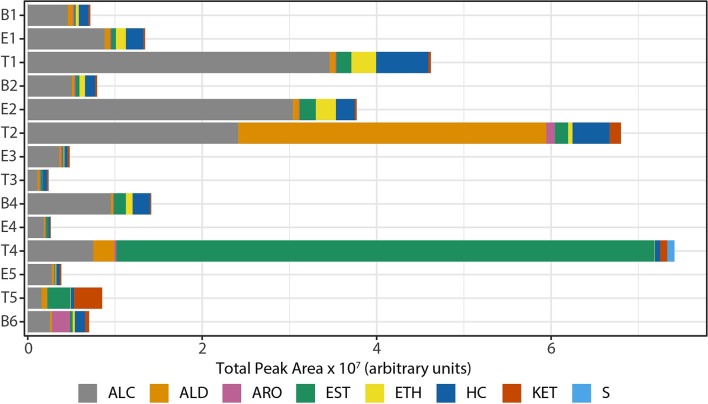
Composition of sputum volatile metabolomes by chemical class, represented in absolute signal intensity. HC, hydrocarbons; ALC, alcohols; ETH, ethers; ALD, aldehydes; KET, ketones; CA/EST, carboxylic acids and esters; ARO, aromatics; S, sulfur-containing; B, baseline; E, exacerbation; T, treatment.

**Figure 6:**
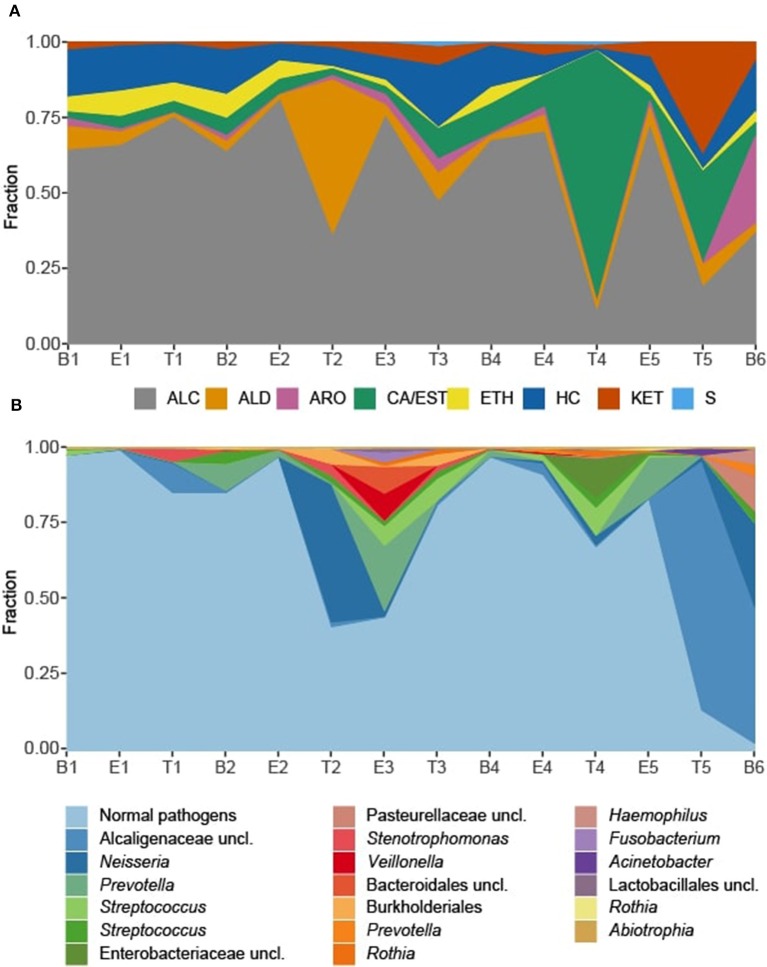
Sputum metabolome and microbiome composition. **(A)** Sputum metabolome composition by chemical class, ordered by time of collection. **(B)** Sputum microbiome composition by OTU. *Escherichia* and *Staphylococcus* reads, representing the “normal pathogens” have been summed to highlight deviations in other taxa of the sputum microbiome. B, baseline; E, exacerbation; T, treatment; HC, hydrocarbons; ALC, alcohols; ETH, ethers; ALD, aldehydes; KET, ketones; CA/EST, carboxylic acids and esters; ARO, aromatics and heteroaromatics; S, sulfurous.

We observed that the total intensity of the volatile metabolite signature (as determined via total peak area) varied from sample to sample (Figure 5), with treatment samples having the highest mean and median metabolite signal intensity (Supplementary Figure 5). The higher signal intensity in treatment samples was correlated with the detection of more compounds in those samples, but the correlation between total peak area and the number of detected compounds was weak (*R*^2^ = 0.48; Supplementary Figure 5). While there was no statistically significant difference in the mean signal intensity by disease state, baseline samples had the least amount of variation across all time points (Supplementary Figure 5). The intensity of the volatile metabolite signals differed across time from B1 to E2 (Figure 5), however the chemical composition of the volatiles varied little, and was dominated by alcohols (Figures 5, 6). At T2 we observed a dramatic shift in the volatile metabolites, with the relative abundance of aldehyde molecules increasing by 100-fold, followed by a return to the same initial chemical composition for specimens collected from E3 to E4. Treatment 4 again had major metabolic shifts, this time due to an increase in two carboxylic acids, octanoic acid and decanoic acid. These two compounds were detected at eight other time points, but at concentrations that were 100-fold to 10,000-fold lower. T4 also had a 10-fold increase in the concentration of aldehydes compared to the preceding four samples. The last two time points, T5 and B6, had entirely unique chemical compositions with a high proportion of ketones in the former and aromatic compounds in the latter. We did not find that disease state correlated to a sputum metabolome chemical composition. Rather, we found that baseline and exacerbation samples were rather uniform in chemical composition and similar to one another, with a relatively high abundance of alcohols, while treatment samples differed from the other two disease states and were highly variable treatment to treatment (Figure 6).

### Associations of Microbes and Metabolites

To explore correlations between the sputum microbiomes and metabolomes, we first evaluated the two data sets for concurrent changes across the samples (Figures 6). While there were significant fluctuations in the relative abundances in *Staphylococcus* and *Escherichia* reads from B1 to E5, for most time points these two genera constituted the majority of the taxa detected in the sputum. The exceptions to this rule were observed at T2 and E3, with the transient increases in *Neisseria* and *Prevotella*, respectively, and T4, with a relative increase in *Streptococcus* and unclassified *Enterobacteriaceae* with a near loss of *Escherichia*. These sputum microbiome fluctuations co-occurred with observable fluctuations in the volatile metabolome composition. Additionally, at T5 and B6 the microbiome composition shifted to dominance by *Alcaligenaceae*, with *Escherichia* and *Staphylococcus* becoming minor constituents. Again, this significant shift in microbiome was reflected in the metabolome, where the previous dominance by alcohols was lost.

**Figure 7:**
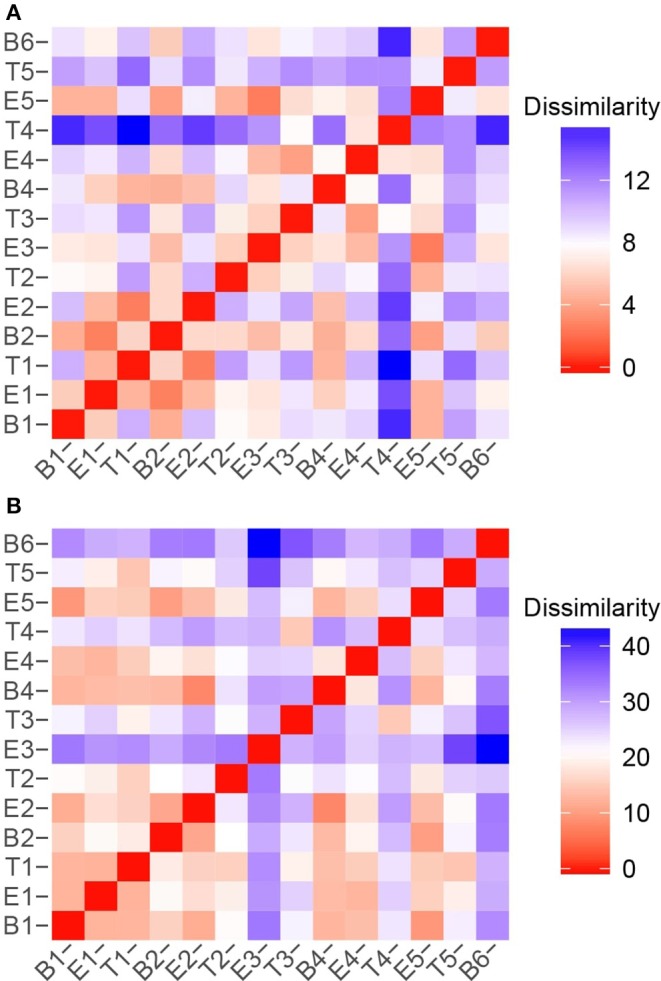
Sample to sample variation represented as dissimilarity matrices using Manhattan distances calculated with unstandardized variables. **(A)** Chemical class relative abundances were used as variables. **(B)** OTU relative abundances were used as variables. The *Staphylococcus* and *Escherichia* reads, representing the study participant's “normal pathogens” were summed to reduce the influence of their stochastic changes, and enhance the influence of the other OTUs on the dissimilarity matrices.

To quantify the similarities and differences between sputum samples, we calculated Manhattan distances based on chemical classes of the metabolomes (Figure 7A) and the microbiome compositions (Figure 7B). Using the relative abundances of chemical classes from the metabolome data, we observe that the sample collected during T4 differs the most strikingly from all preceding samples (Figure 7A). The chemical composition of E5 returned to a state that was similar to early baseline and exacerbation samples, followed by another significant chemical shift in T5. To analyze the microbiome data, we reduced the influence of the stochastic shifts in the proportions of *Staphylococcus* vs. *Escherichia* reads—i.e., the study participant's “normal pathogens”—by summing those reads prior to calculating the distances. The result is that differences in the relative proportions of the other 21 most abundant OTUs are emphasized. We observed that the microbiome of sputum sample E3 showed the first major shift in composition, and was unlike any other samples collected. This correlated to our observation that the microbiome of E3 had the highest alpha diversity. The high microbiome diversity was temporary, however, with samples B4 and E4 resembling early samples due to the returning dominance of the normal pathogens.

**Figure 8:**
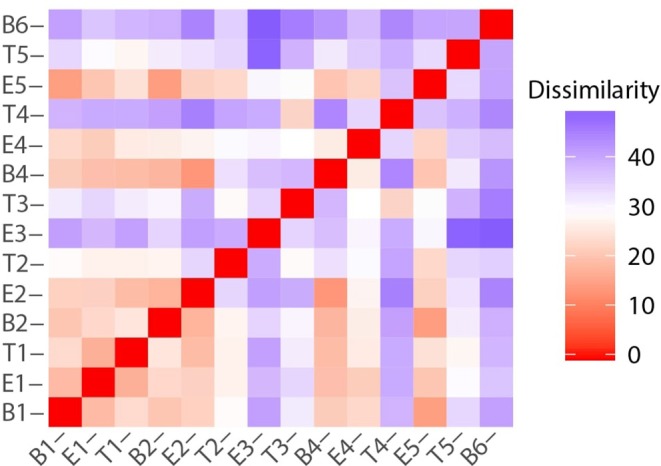
Sample to sample variation represented as a dissimilarity matrix using Manhattan distances calculated with chemical class abundance and proportions of the top 23 most abundant OTUs as standardized variables.

While there were unifying features in the dissimilarity matrices constructed using microbiome and metabolome data (e.g., the uniqueness of T4), they were not fully synchronized. However, we posited that combining the data sets would present a more holistic picture of this study participant's lung disease, and therefore we calculated the dissimilarities between samples using a concatenated microbiome and metabolome data set (Figure 8). We again observe that sputum samples B1 through E2 were highly similar, which was followed by a sustained period of variance. With the exception of B4 and E4, when comparing any pair of proximate samples from T2 to B6, their dissimilarities were higher than the median, largely driven by the uniqueness of samples collected during treatment periods. While B1, B2, and B4 baseline samples all had similar microbiome-metabolome compositions (i.e., higher than median similarities), we observe that the last specimen, B6, is indeed distinctly different from every other sample previously collected, baseline or otherwise.

**Figure 9:**
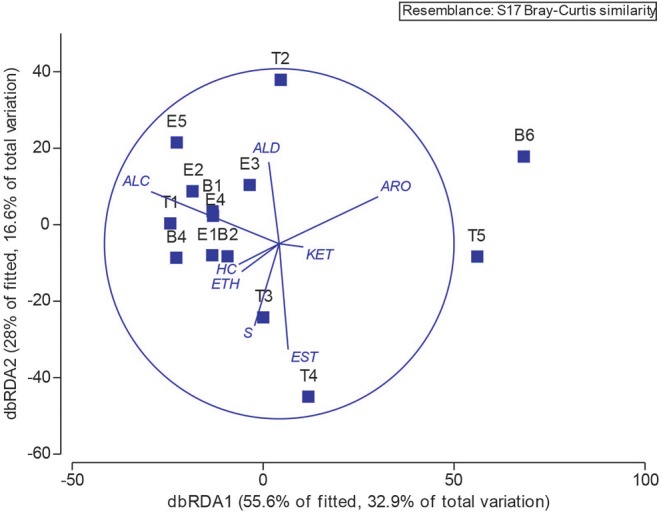
Operational taxonomic unit and chemical class overlay. Distance-based linear model recapitulates the relationship between the airway microbiomes in each sample. Superimposed are vectors showing which chemical classes are best correlated with the microbial communities. Length and direction of vectors correspond to the strength of the association between the metabolite and the microbial communities. B, baseline; E, exacerbation; T, treatment. HC, hydrocarbons; ALC, alcohols; ETH, ethers; ALD, aldehydes; KET, ketones; EST, carboxylic acids and esters; ARO, aromatics and heteroaromatics; S, sulfurous.

Next, we explored the potential associations of specific microbes to individual metabolites by performing Spearman and Pearson Rank analyses between 16S OTU abundances and metabolite signal intensities. We found no statistically-significant correlations, whether we binned all metabolites that are known to be associated with *E. coli* or *S. aureus*, the two best-studied bacterial volatile metabolomes represented in this sample set (Allardyce et al., 2006; Filipiak et al., 2012; Tait et al., 2014; Baptista et al., 2019; Jenkins and Bean, 2019), or treated metabolites independently, e.g., correlated *E. coli* relative abundance to indole absolute or relative abundance. Lastly, we returned to the chemical classification data, and examined the relationship between bacteria and metabolome composition with a distance-based linear model (Figure 9). The position of each point in Figure 9 is dictated by the Bray-Curtis similarity of the airway microbiome composition in each sample, and the superimposed vectors showing which chemical classes are best correlated with the microbial communities. From this view, we observe that the highly similar microbiome compositions of samples B1—E2, B4, E4, and E5, characterized by dominance with the study participant's normal pathogens (i.e., *Staphylococcus* or *Escherichia*), are highly associated with a higher relative abundance of alcohols. Interestingly, T3 and T4 were also dominated by *Staphylococcus*, but did not cluster with the other Staph-dominated samples. The microbiome composition in these specimens does contain more *Streptococcus* spp., and is strongly associated with an increase in sulfur-containing compounds and carboxylic acids/esters in T3 and T4, respectively. We observed that sample T2, characterized by its high abundance of *Neisseria*, is correlated with increased detection of aldehydes. The distinctly clustering outlier samples T5 and B6, dominated by *Achromobacter*, correlated with a shift toward more oxidized compounds (aromatics, ketones) and away from hydrocarbons and alcohols.

## Discussion

Prior studies of both the microbiomes and metabolomes in the CF lung have suggested high inter-person variability, which can be greater than the variability identified between clinical states, thereby limiting broad extrapolation of research findings to the general CF population (Carmody et al., 2013; Quinn et al., 2016). A cross-sectional study comparing paired baseline and exacerbation samples found some persons with CF exhibiting little change and other persons with dramatic changes in bacterial community structure (Carmody et al., 2013). A study of the LC-MS derived metabolome capturing larger and more polar molecules (e.g., immune lipids) in the sputum of 11 individuals with CF found that the individual source had a larger influence than clinical state, with only 12% of over 4,000 identified metabolites being unique to pulmonary exacerbation samples (Quinn et al., 2016). These findings support a personalized approach to identifying microbiome and metabolomic changes consistent with changes across clinical states.

With this study, we sought to investigate changes in both the airway microbiome and volatile metabolome of a single person with CF over a 12 month period of recurrent pulmonary exacerbations. We found that although the study participant retained a set of core bacteria that dominated the bacterial community during most baseline and exacerbation samples (specifically *Escherichia coli* and *Staphylococcus aureus*), a transition to a new dominant bacterium (*Achromobacter xylosoxidans*) occurred after the study participant's fifth antibiotic course. This new bacterium continued to grow in subsequent respiratory cultures for at least 6 months following the study. Interestingly, this change in dominant bacterium did not seem to correlate with a change in treatment antibiotics used, as similar IV and oral antibiotics were used throughout the 12 month time period. Alpha diversity measures were also not associated with the transition in dominant organism. When examining community bacterial structure using both PCoA and Random Forest, we found that treatment samples tended to cluster together, albeit not significantly. One bacterial phylum (Bacteroidetes) and one bacterial genus (*Stenotrophomonas*) were significantly more abundant in treatment samples compared to baseline and exacerbation samples using two different analysis measures (Random Forest and DESeq2). While our Random Forest analysis had a high OOB estimation of error, it is worth noting that with repeated assessments of the same data set, Bacteroidetes and *Stenotrophomonas* remained the most important variables and treatment samples were always differentiated from baseline and exacerbation samples in the proximity plots.

Similar to our results, prior longitudinal studies of the CF airway have not always found a significant change in the relative abundance of bacteria to be associated with exacerbation onset (Whelan et al., 2017; Sherrard et al., 2019). While we found rather consistent differences in both bacterial relative abundance and volatile metabolites when comparing treatment samples to baseline and exacerbation samples, this has not always been observed in other studies. One study found no consistent differences in Shannon diversity across stable, intermediate, or treatment samples over time, even though diversity was always fluctuating (Whelan et al., 2017). Some studies have found increases in fermentative anaerobes are associated with the onset of exacerbation (Tunney et al., 2011; Carmody et al., 2013, 2018). Another study obtaining enhanced cultures longitudinally found more constant communities in stable persons with CF, while those with frequent exacerbations had higher variability in their community structures (Sherrard et al., 2019). As our study participant experienced multiple exacerbations during the study, this may explain, in part, the changing relative abundance and alpha diversity we saw.

A deeper analysis of one baseline and two exacerbation samples confirmed that *E. coli* and *S. aureus* were the dominant bacteria within the airway, but the relative abundance was different when analyzed via 16S and shotgun sequencing. This difference is likely multifactorial, including PCR amplification bias associated with 16S rDNA sequencing, potential misclassification bias from human DNA contamination, and differences in reference databases used for sequence alignment (Hahn et al., 2018b). However, with shotgun sequencing we were also able to explore additional characteristics potentially important to clinical response, including the presence of bacteriophages and antibiotic resistance genes.

Prior metagenomic studies of the CF airway have found that the majority of DNA viruses identified were bacteriophages (Moran Losada et al., 2016). In our study, we had similar findings; more than 70% of the viral reads were attributed to bacteriophages, and most were associated with Enterobacteria, *Escherichia*, and *Staphylococcus*. These phages can have important clinical impacts. First, filamentous phages associated with *E. coli* have been shown to promote biofilm formation (Secor et al., 2015). Second, *S. aureus* bacteriophages in the CF airway were associated with genomic alterations of the bacterium, likely passing on virulence traits and perhaps enhancing its ability to survive despite antibiotic pressures (Goerke et al., 2004). Lastly, bacteriophages in general have been shown to transfer antibiotic resistance, such as efflux pumps, beta-lactam resistance, and fluoroquinolone resistance, particularly within the CF lung (Fancello et al., 2011; Brown-Jaque et al., 2018).

In our evaluation of antibiotic resistance detected by shotgun sequencing, we found that the results were generally consistent with the clinical cultures, and more sequences aligned to antibiotic resistance genes in the two exacerbation samples. One caveat is that the potential for MRSA was detected by sequencing in 2 of 3 samples where the corresponding cultures grew MSSA; this signifies the limitations of making antibiotic decisions on clinical culture results alone. Furthermore, prior studies have shown that multidrug antibiotic resistance is associated with decreased alpha diversity, the presence of *Achromobacter* species, and lower pulmonary function (Bacci et al., 2017; Hahn et al., 2018c). Several studies utilizing shotgun sequencing to analyze the CF airway have also found a congruence of antibiotic resistance detected in clinical cultures, and an association of antibiotic resistance with *E. coli* and *A. xylosoxidans* (Lim et al., 2014; Bacci et al., 2017; Feigelman et al., 2017). Importantly, genes encoding resistance to antibiotics not recently used can be present, suggesting that the development of antibiotic resistance in the CF lung is partly related to direct antibiotic pressures, partly through the presence of multidrug efflux pumps, and partly through the presence of mobile elements encoding resistance that are carried in bacteriophages as mentioned earlier (Fancello et al., 2011; Lim et al., 2014; Brown-Jaque et al., 2018).

The primary goals of the study were to identify microbiome and metabolome characteristics indicative of clinical state (baseline, exacerbation, and treatment) and to evaluate intra-person variability between clinical disease states. We observed that the baseline and exacerbation specimens did not significantly differ in either their microbiome or metabolome, but that treatment specimens T2-T5 and the final baseline sample (B6) were dissimilar from the rest. However, because the sputum characteristics of these samples are all unique, we are unable to identify specific microbiome or metabolome signatures of treatment, nor determine definitive associations between the microbiome and metabolome compositions we observed. Additionally, the common trait of these specimens' microbiomes is a reduction in the study participant's normal pathogens and concomitant increase in other taxa, but published data on the volatile metabolomes of bacteria other than *P. aeruginosa, S. aureus, E. coli*, and *Mycobacteria* spp. are relatively scarce. The dominance of the T4 metabolome by octanoic and decanoic acids co-occurred with the highest relative abundance of *Streptococcus* and *Rothia* in any sample. Generally, though, mid-chain fatty acids are characterized as antibacterial against a broad range of Gram positive and Gram negative pathogens (Desbois and Smith, 2010), and free and derivatized forms of octanoic and decanoic acids have been shown to inhibit *Staphylococcus* spp., *Streptococcus* spp., *Neisseria gonorrhoeae*, and *E. coli* growth (Kabara et al., 1972; Bergsson et al., 1999; Skrivanová et al., 2008). It is also feasible that these two compounds are associated with treatment rather than infection, as decanoic acid is commonly used as a solubilizing agent in medications, and mid-chain fatty acid esters are common prodrugs used to improve lipophilicity.

In the baseline and exacerbation samples, we observed large variations in the relative abundances of *S. aureus* and *E. coli* 16S amplicon sequencing reads, however, we did not find correlated fluctuations in volatile metabolites that are produced by these bacteria. *In vitro* experiments have demonstrated that both *E. coli* and *S. aureus* produce volatile alcohols (Allardyce et al., 2006). Therefore, one possible explanation is that the production of alcohols by *S. aureus* and *E. coli* are similar in the environment of the CF lung, and aggregating the metabolome into chemical classes removes the species-specific correlations we would otherwise observe. However, we did attempt to find correlations between *S. aureus* or *E. coli* reads and the abundances of their known metabolites, and failed to do so. Interestingly, we did observe reductions in the relative abundance of alcohols when the relative abundance of the normal pathogens decreased, consistent with the reduced alcohol production observed when these organisms are dosed with antibiotics above minimum inhibitory concentrations *in vitro* (Allardyce et al., 2006). It is also possible that the VOCs observed were indicative of clinical state rather than correlating directly with the abundance of an individual species, perhaps due to variations in functional genetics and/or expression of bacterial genes. This would still provide a useful tool that could be translated into clinical practice, but requires further study.

By integrating both the metabolome and microbiome data, we observed an overall smoothing of the sputum characteristics that help to reveal time/treatment-dependent patterns. While changes in the sputum did not clearly correlate with clinical disease state (i.e., predict exacerbation onset), they did correlate with clinical culture data and revealed when the character of the lung environment was and was not changing. The microbiome and metabolome data also suggested that there was a fundamental shift in the study participant's lung disease during and after the 5th treatment, which correlated to culture results; beginning with B6, and for at least 6 months afterward, the subject consistently cultured *A. xylosoxidans*, and *E. coli* was not detected again.

Limitations to the study include the translational capacity of a longitudinal study design from a single person with CF. However, the inter-individual variability in the CF airway microbiome is often high, frequently influenced by the dominant organism which may be different between study participants, and can lead to limitations in interpreting results due to that variability (Boutin et al., 2015). Thus, understanding intra-person variability across clinical states can inform future study design in larger cohorts (Caverly et al., 2019; Hahn and Zemanick, 2019). Furthermore, n-of-1 studies can still provide important data to the literature, and may provide a way forward for using the CF microbiome and metabolome in individualized medicine (Lillie et al., 2011). Moving forward into larger cohorts, it may be necessary to look at changes between each study participant as a series of n-of-1 studies instead of averaging species relative abundance, diversity measures, etc., to truly begin to bring these findings into meaningful clinical practice. By itself, this study shows that antibiotic treatment has the largest impact on taxonomic relative abundance and the metabolome, and there may be little dissimilarity between baseline and exacerbation in a CF person experiencing frequent pulmonary exacerbations. Another limitation was the lack of inclusion of WGS data for treatment samples, as these time points showed the most variability in our cohort. Unfortunately, we were limited in the amount of bacterial DNA available and were only able to perform WGS on baseline and exacerbation samples.

In summary, we found that using a combination of metabolome and microbiome data, we could corroborate changes in community structure and metabolism in the lung environment over the span of a year in a subject with unstable lung disease, and these data correlated with short-term and long-term clinical culture data. For this particular dataset, the greatest amount of change was associated with antibiotic treatment. Future directions include performing a similar analysis of the association between the bacterial microbiome and volatile metabolites in a larger cohort. The results from this study will allow us to incorporate the level of intra-person variability observed so that we may be able to detect a potential signature that could be utilized in identifying transitions between clinical states.

## Data Availability Statement

The sequence dataset supporting the conclusions of this article is available in the NCBI SRA repository under BioProject PRJNA437613 (16S sequencing data) and PRJNA515276 (trimmed and human filtered whole genome shotgun sequencing data). For PRJNA437613, the sample IDs are as follows: B1 AHCF01a, B2 AHCF01d, B4 AHCF01d3, B6 AHCF01d5, E1 AHCF01b, E2 AHCF01b2, E3 AHCF01b3, E4 AHCF01b4, E5 AHCF01b5, T1 AHCF01c, T2 AHCF01c2, T3 AHCF01c3, T4 AHCF01c4, and T5 AHCF01c5.

## Ethics Statement

The studies involving human participants were reviewed and approved by Children's National Health System. Written informed consent to participate in this study was provided by the participants' legal guardian/next of kin.

## Author Contributions

AH designed the study and obtained and maintained IRB approval. IS and AK were responsible for identifying eligible study subjects and obtaining consent. AH, HB, KW, RF, and KC designed the study experiments and determined the appropriate bioinformatic and statistical approaches for analyzing the data. AH, HB, TD, JP, and KW were responsible for data collection and analysis. AH and HB wrote the text of the manuscript. AH, HB, KW, TD, and JP generated the manuscript figures. All authors provided critical review of the manuscript and approved of the final manuscript as written.

## Conflict of Interest

The authors declare that the research was conducted in the absence of any commercial or financial relationships that could be construed as a potential conflict of interest.
