# Supplementary material for: Longitudinal Associations of the Cystic Fibrosis Airway Microbiome and Volatile Metabolites: A Case Study
Source: Front Cell Infect Microbiol. 2020 Apr 28;10:174. doi: 10.3389/fcimb.2020.00174 (PMC7198769; doi:10.3389/fcimb.2020.00174)
Supplement: Supplementary file 1 [file Data_Sheet_1.docx]

Supplementary Material

# Supplementary Data

**1.1 Shotgun Whole Genome Sequencing**

Three samples (B1, E3, and E5) also underwent shotgun whole genome sequencing (WGS), allowing for additional analyses. KneadData was used to remove human contaminant sequences, resulting in the following remaining sequencing reads: B1 4,730,384; E3 17,874,465; E5 9,272,526. PathoScope 2.0 was used to determine bacterial taxonomic abundance and the presence of bacteriophages and viruses. A mean of 55% of remaining reads were assigned to a bacterial or viral taxa (B1 1,787,076, 37.8%; E3 12,826,379, 71.8%; E5 5,161,821, 55.7%). Bacterial taxonomic abundance at the strain level is shown in Supplementary Figure 1. Interestingly, the percent of bacterial reads attributed to *E. coli, S. aureus,* and *A. xylosoxidans* were not significantly similar to what was identified using 16S rRNA sequencing, with the largest discrepancy noted in the B1 sample (see Supplementary Table 1).

We also found 30 different types of bacteriophages within these samples, which are shown in Supplemental Figure 3. The reads associated with bacteriophages were a mean of 0.54% of the total assigned reads (range 0.47% to 0.66%) and mean of 71.2% of total viral reads (range 37.5% to 81.1%). As expected, the majority of the bacteriophages identified were associated with *Staphylococcus* (n=15, 50%) and Enterobacteria/*Escherichia* (n=12, 40%), which were the predominant bacteria within the microbiome community. Further, the trends in relative abundance of *Staphylococcus* vs. Enterobacteria/*Escherichia* bacteriophage reads (B1 < E3 < E5) correlate with the *S. aureus* vs. *E. coli* relative abundances observed by WGS.

We next evaluated the presence of antibiotic resistance genes, focusing on those antibiotics commonly used to treat lung infections in cystic fibrosis patients. These included aminoglycosides (e.g., tobramycin), beta-lactams (e.g., ceftazidime), fluoroquinolones (e.g., ciprofloxacin), glycopeptides (e.g., vancomycin), lipopeptides (e.g., colistin), macrolides (e.g., azithromycin), rifampin, sulfonamides (e.g., trimethoprim-sulfamethoxazole), tetracyclines (e.g., doxycycline), and trimethoprim. The distribution of the percentage of sequence reads mapped to bacterial genes affecting these antibiotic classes is shown in Supplemental Figure 4. Overall, the percentage of sequencing reads mapped to an antibiotic resistance gene were higher in the exacerbation samples than the baseline sample (B1 32,698, 0.63%; E3 424,115, 2.11%; E5 157,383, 1.51%; p = 0.001). The majority of antibiotic resistance genes identified were due to multi-drug resistance mechanisms (e.g., porins and efflux pumps), while the next most common were aminoglycosides, beta-lactams, and fluoroquinolones. These findings corroborated the antibiotic resistance recognized in the corresponding clinical cultures. The B1, E3, and E5 cultures all grew an *E. coli* which had an extended spectrum beta-lactamase and was also resistant to gentamicin/tobramycin and ciprofloxacin/levofloxacin. Interestingly, although the B1 and E5 cultures grew MSSA compared to the MRSA that grew in the E3 culture, all three samples had sequences that mapped to the penicillin binding protein (PBP) (B1 81% beta-lactamases, 19% PBP; E3 78% beta-lactamases, 22% PBP; E5 64% beta-lactamases, 36% PBP). The E5 culture also grew an *A. xylosoxidans* that was resistant to all aminoglycosides and all cephalosporins except for ceftazidime (consistent with their intrinsic resistance) and ciprofloxacin.

# Supplementary Figures and Tables

## Supplementary Figures


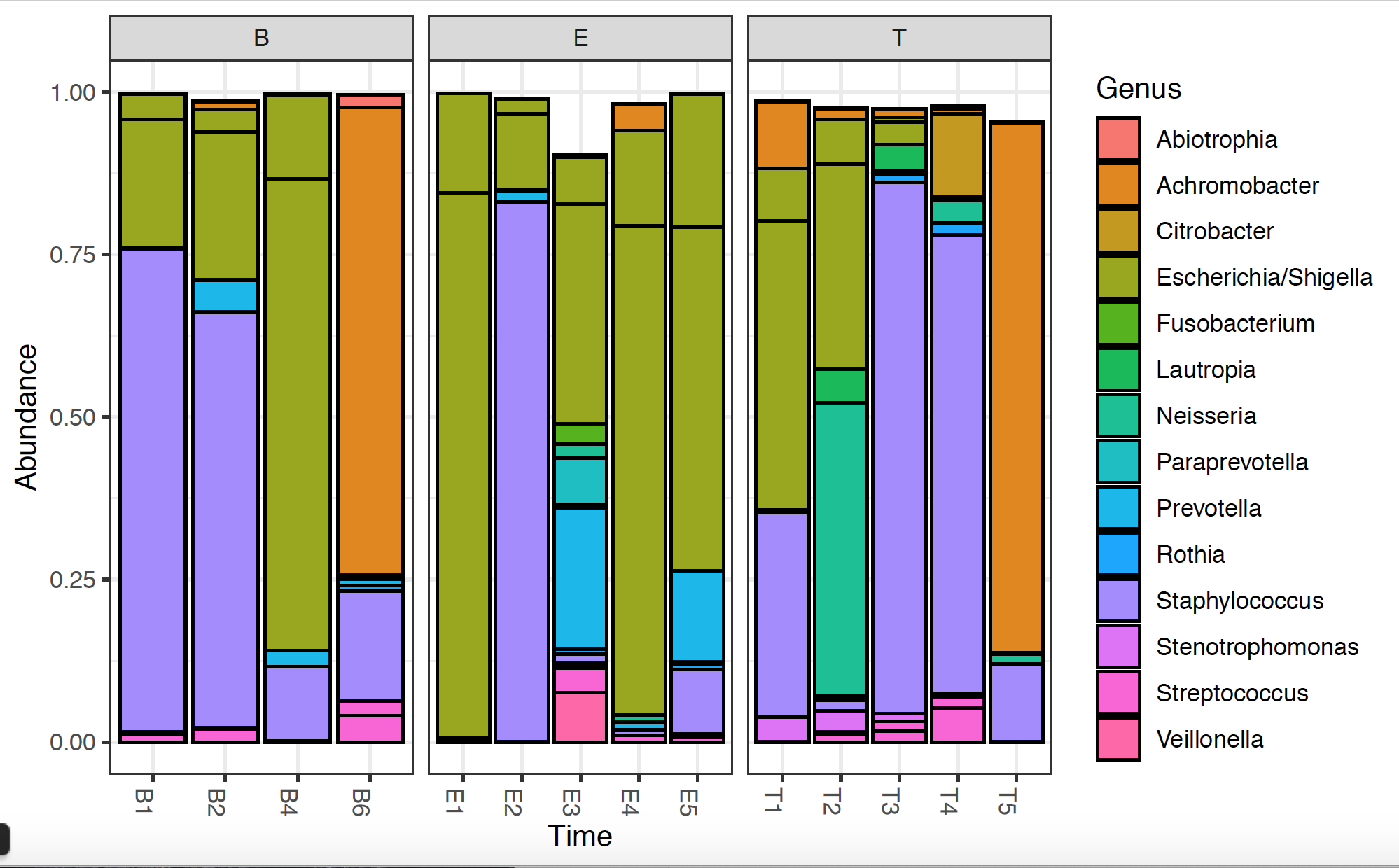


**Supplementary Figure 1. Relative taxonomic abundance.** Relative abundance as determined by amplicon sequencing variants (ASV). The top 20 ASVs are included. B, Baseline; E, Exacerbation; T, Treatment.

**
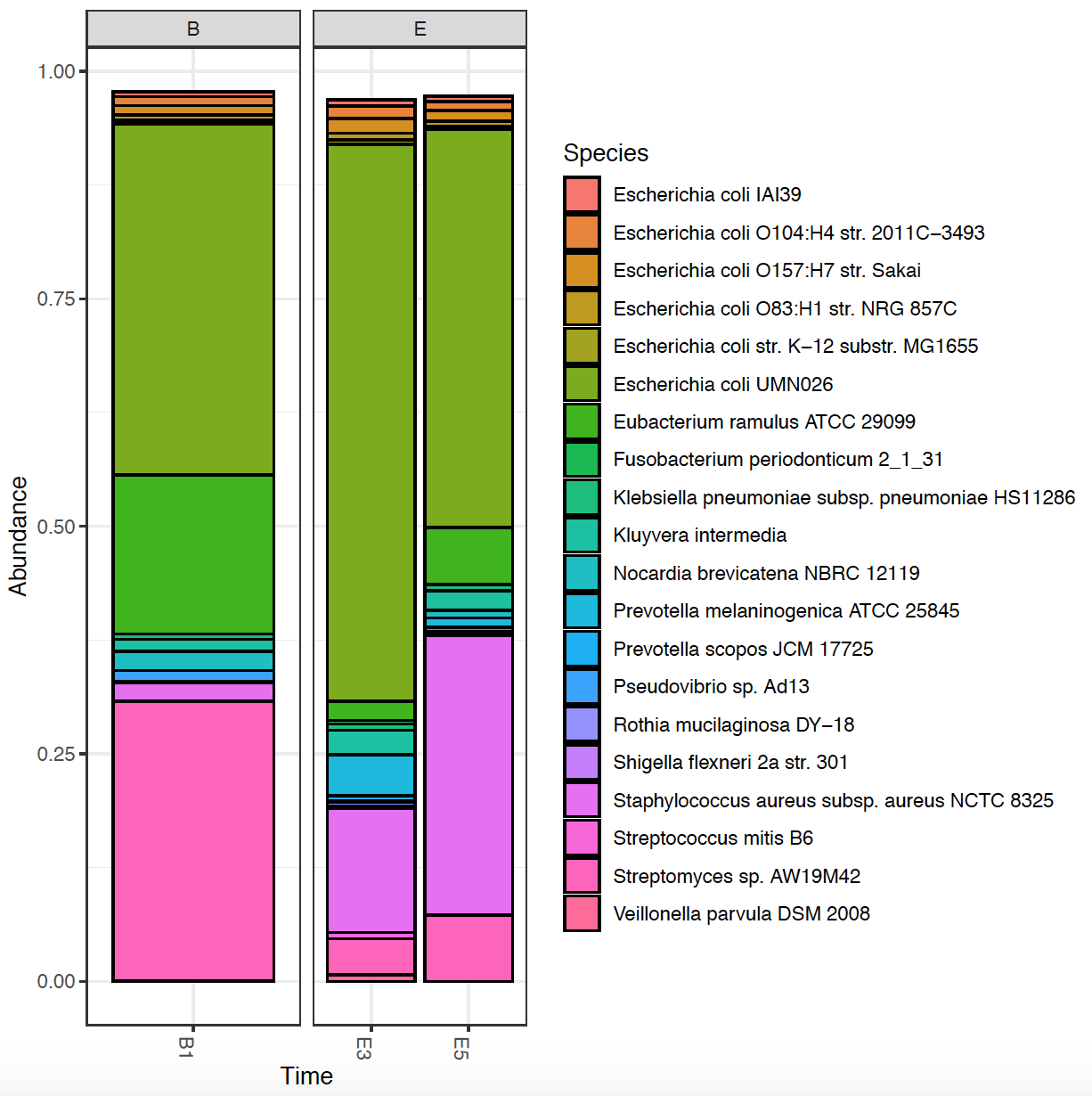
**

**Supplementary Figure 2. Bacterial taxonomic abundance via PathoScope.** Relative abundance of bacterial species as determined by shotgun whole genome sequencing. The top 20 species are included. The relative abundance was based on the following number of reads: B1 1,633,515; E3 12,625,785; E5 4,974,985. B, Baseline; E, Exacerbation.


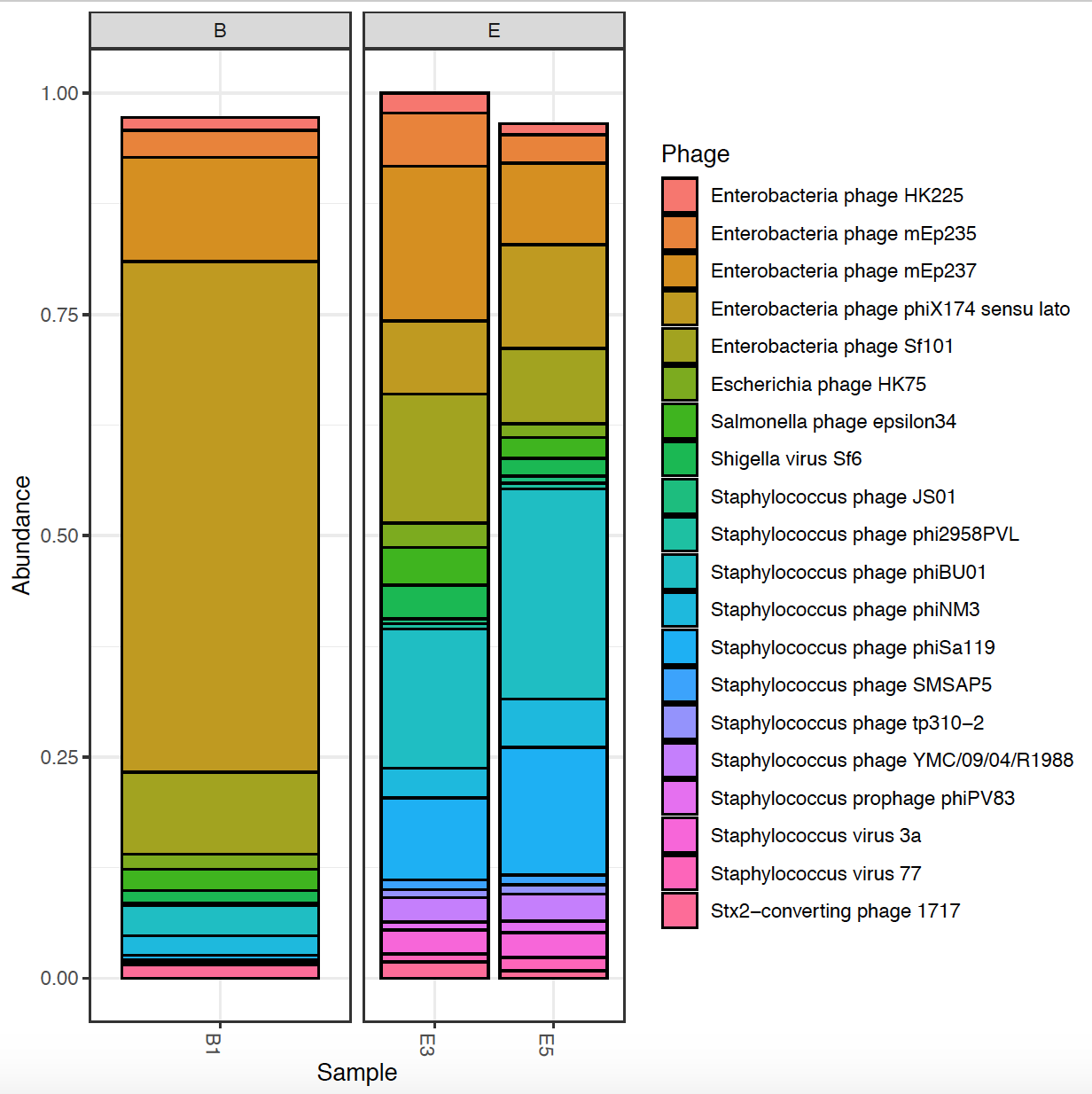


**Supplementary Figure 3. Bacteriophages identified via PathoScope.** Relative abundance of bacteriophages as determined by shotgun whole genome sequencing. The top 20 bacteriophages are included. The total number of sequences aligned to bacteriophages were as follows: B1 8,853; E3 59,862; E5 34,164. B, Baseline; E, Exacerbation. Blue Enterobacteria/*Escherichia;* Orange *Staphylococcus.*


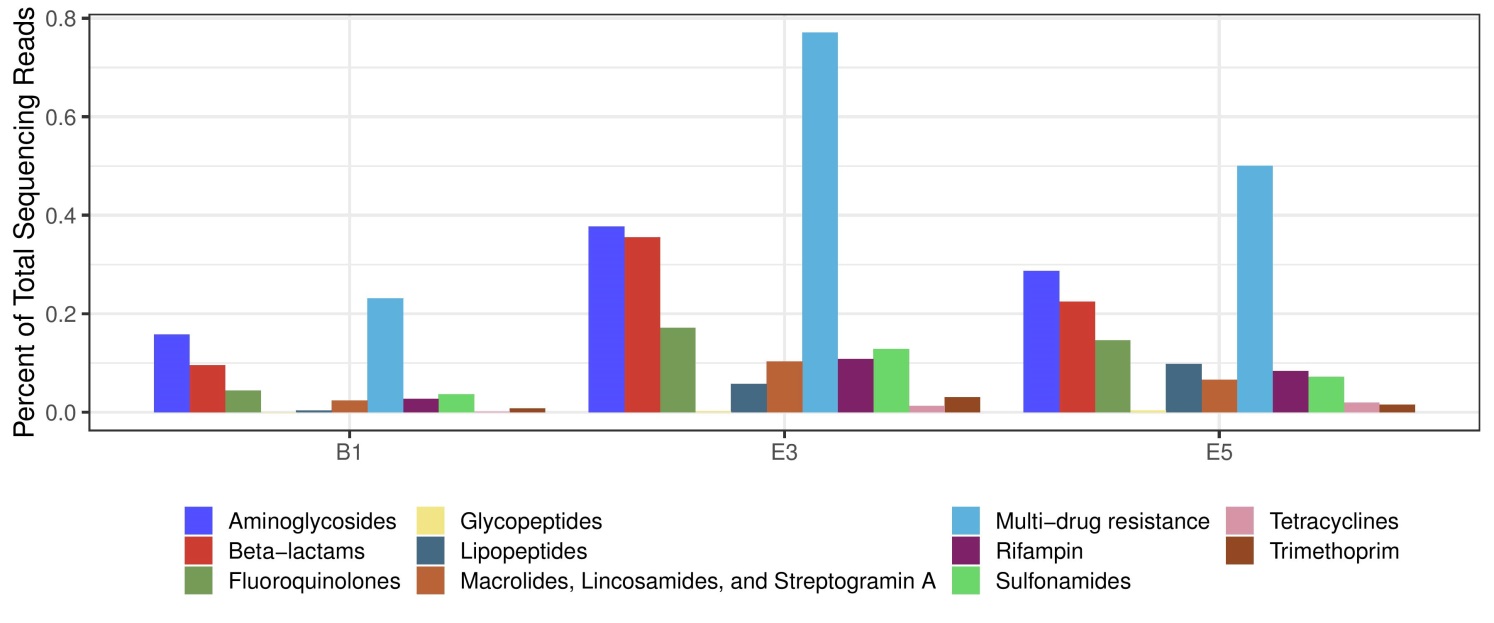


**Supplementary Figure 4. Antibiotic resistance genes identified via AmrPlusPlus.** The percent was based on the following number of total sequencing reads: B1 4,730,384; E3 17,874,465; E5 9,272,526. B, Baseline; E, Exacerbation.


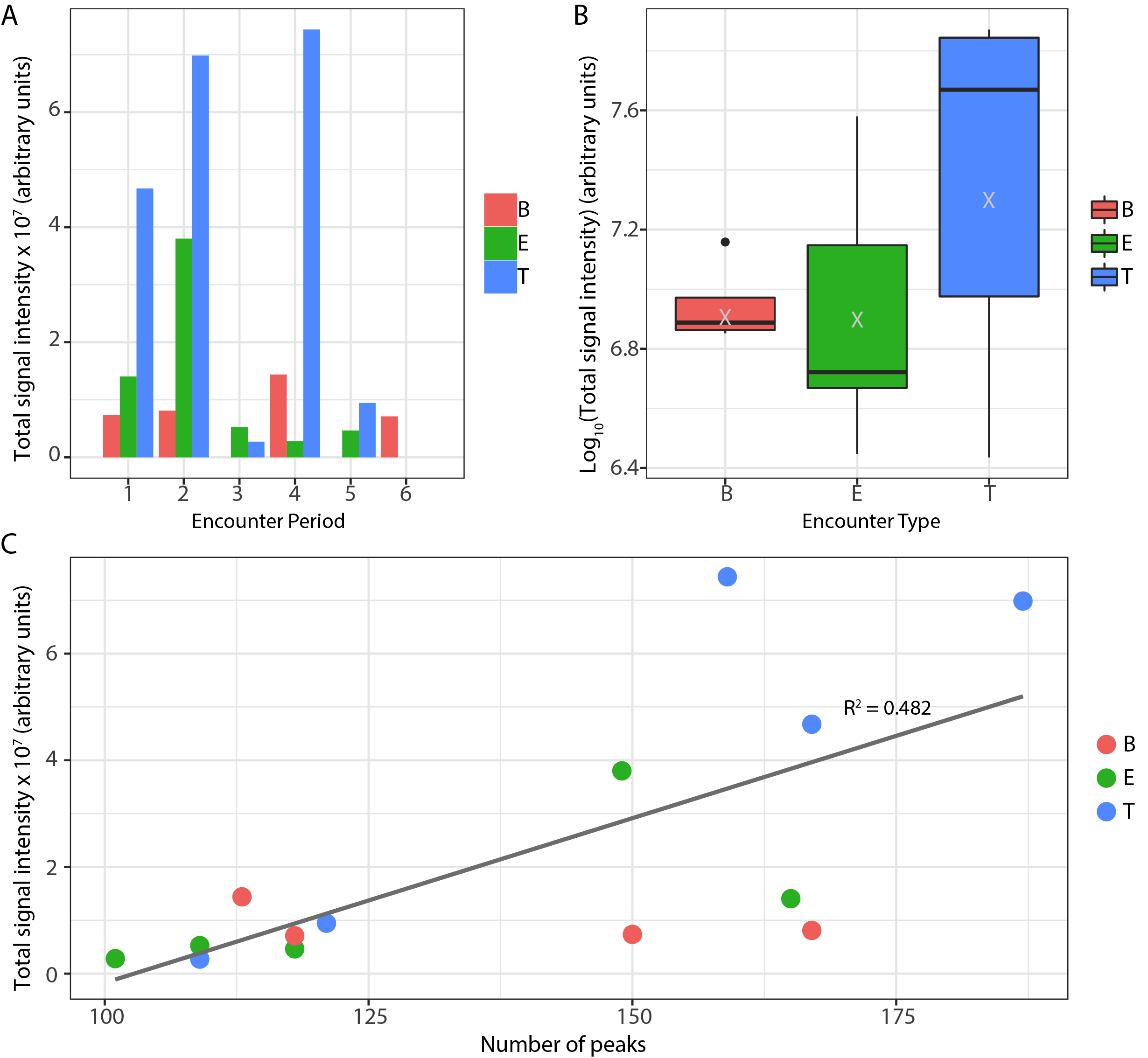


**Supplementary Figure 5. Signal Intensity of Volatile Organic Compounds Detected.** Panel A. Total signal intensity of all compounds measured for each sample. B, baseline (red); E, exacerbation (green); T, treatment (blue). Missing samples (i.e., B-3, B-5, B-6, T-6) were not treated as zeros in the subsequent analyses. Panel B. Interquartile ranges of signal intensity by disease state, log_10_ scale. Mean signal intensity indicated by a cross mark (X). Panel C. Total signal intensity versus the number of compounds detected for each sample.

## Supplementary Tables

**Supplementary Table 1. Comparison of relative abundance of top three bacterial species detected by 16S OTU, 16S ASV, and shotgun whole genome sequencing.**

|  |  | *Escherichia/*  *E. coli* (all strains) | *Staphylococcus/*  *S. aureus* | *Achromobacter/*  *A. xylosoxidans* | R2 | P value |
| --- | --- | --- | --- | --- | --- | --- |
| B1 | 16S OTU* | 24.4% | 72.8% | 0% | Reference | Reference |
|  | 16S ASV^†^ | 23.6% | 74.3% | 0% | 0.999 | 0.012 |
|  | WGS^‡^ | 42.1% | 2.0% | 0% | 0.021 | 0.907 |
| E3 | 16S OTU | 41.0% | 1.1% | 0.2% | Reference | Reference |
|  | 16S ASV | 41.1% | 1.4% | 0.3% | 1 | 0.002 |
|  | WGS | 66.1% | 13.6% | 0.2% | 0.969 | 0.113 |
| E5 | 16S OTU | 73.4% | 9.8% | 0.2% | Reference | Reference |
|  | 16S ASV | 73.3% | 9.8% | 0.2% | 1 | <0.001 |
|  | WGS | 47.4% | 30.7% | 0.1% | 0.708 | 0.363 |

*16S OTU, 16S rRNA sequencing using 97% similarity to determine operational taxonomic units. Percentages based on 11,413 total bacterial reads for B-1, E-3, and E-5.

^†^16S ASV, 16S rRNA sequencing using amplicon sequencing variants. Percentages based on 55,573 ASVs for B1, 26,939 ASVs for E3, and 31,006 ASVs for E5.

^‡^WGS, shotgun whole genome sequencing. Percentages based on 1,633,515 total bacterial reads for B1, 12,625,785 total bacterial reads for E3, and 4,974,985 total bacterial reads for E5.
